# Cytotoxic effects of ivermectin on *Giardia lamblia*: induction of apoptosis and cell cycle arrest

**DOI:** 10.3389/fmicb.2024.1484805

**Published:** 2024-10-31

**Authors:** Florencia Nicole Barzola, Jerónimo Laiolo, Camilo Cotelo, Mariana Belén Joray, Ximena Volpini, María Romina Rivero, Andrea Silvana Rópolo, María Carolina Touz, Constanza Feliziani

**Affiliations:** ^1^Instituto de Investigación Médica Mercedes y Martín Ferreyra (INIMEC), Consejo Nacional de Investigaciones Científicas y Técnicas (CONICET), Universidad Nacional de Córdoba, Córdoba, Argentina; ^2^Facultad de Ciencias de la Salud, Universidad Católica De Córdoba, Córdoba, Argentina; ^3^Centro de Investigación y Desarrollo en Inmunología y Enfermedades Infecciosas (CIDIE), Consejo Nacional de Investigaciones Cientí-ficas y Técnicas (CONICET)/Universidad Católica de Córdoba (UCC), Córdoba, Argentina; ^4^Centro de Investigaciones en Bioquímica Clínica e Inmunología – Consejo Nacional de Investigaciones Científicas y Técnicas (CIBICI-CONICET), Córdoba, Argentina; ^5^Instituto De Desarrollo Agroindustrial y De La Salud (IDAS-CONCIET), Universidad Nacional De Rio Cuarto, Rio Cuarto, Argentina

**Keywords:** ivermectin, parasitic infections, cytotoxicity, apoptosis, necrosis, anti-giardial agent

## Abstract

**Introduction:**

*Giardia lamblia* is a flagellated protozoan parasite causing giardiasis, a common intestinal infection characterized by diarrhea, abdominal cramps, and nausea. Treatments employed to combat this parasitic infection have remained unchanged for the past 40 years, leading to the emergence of resistant strains and prompting the search for new therapeutic agents.

**Methods:**

This study investigated the cytotoxic effects of ivermectin (IVM) on *G. lamblia* trophozoites. We conducted dose-response experiments to assess IVM-induced cytotoxicity. We utilized various biochemical and ultrastructural analyses to explore the underlying mechanisms of cell death, including reactive oxygen species (ROS) production, DNA fragmentation, cell cycle arrest, and apoptosis markers.

**Results:**

Our findings demonstrate that IVM induces dose-dependent cytotoxicity and triggers cell death pathways. We found that IVM treatment generates elevated levels of reactive oxygen species (ROS), DNA fragmentation, and arrests of trophozoites in the cell cycle’s S phase. Additionally, ultrastructural analysis reveals morphological alterations consistent with apoptosis, such as cytoplasmic vacuolization, chromatin condensation, and tubulin distribution.

**Discussion:**

The insights gained from this study may contribute to developing new therapeutic strategies against giardiasis, addressing the challenge posed by drug-resistant strains.

## Introduction

*Giardia lamblia*, also known as *G. intestinalis* and *G. duodenalis*, is a widespread intestinal parasite causing giardiasis, impacting approximately 200 million people globally, with a significant prevalence in Asia, Africa, and Latin America. This parasitic infection is transmitted through the fecal-oral route, involving contact with infected individuals, livestock, wild animals, or consuming contaminated water or food containing cysts (Adam, 2021; Farthing, 1997; Gillin et al., 1996). The spectrum of infection ranges from asymptomatic cases to chronic illnesses, presenting symptoms like diarrhea, dehydration, abdominal distension, nausea, vomiting, bloating, and malabsorption. Chronic consequences include fatigue, irritable bowel syndrome, and growth delay in young children (Farthing, 1997; Gillin et al., 1996). In the environment, *G. lamblia* is encased in an outer shell (cyst wall) that enables it to survive outside the host and renders it tolerant to disinfection by hypochlorite (Adam, 2021). Inside the host, cysts differentiate into trophozoites that colonize the intestine and cause disease symptoms.

Existing antiparasitic drugs, such as metronidazole (MTZ), exhibit undesirable side effects and resistance mechanisms, prompting the search for novel, more effective, and safer agents with selective toxicity for the parasitic organism and minimal impact on the host (Upcroft and Upcroft, 2001). A previous study from our laboratory revealed that ivermectin (IVM) has a lethal effect on *G. lamblia* trophozoites at high concentrations and influences the trophozoites differentiation into cysts (Mayol et al., 2020). IVM, a macrolide antiparasitic drug family member, is recognized for its composition, featuring a 16-membered ring derived from avermectin (Caly et al., 2020; Campbell, 2012). Originally approved by the FDA for human use in 1978, IVM—often referred to as a “wonder drug” akin to penicillin and aspirin—has made a profound impact on global health (Sulik et al., 2023). Notably effective in treating parasitic diseases such as river blindness, elephantiasis, and scabies, it earned its discoverers, Satoshi Omura and William C. Campbell, the Nobel Prize in Physiology and Medicine in 2015 (McKerrow, 2015).

As the challenge of parasites resistant to conventional treatments continues to grow, IVM emerges as a multifaceted solution in combating a range of parasitic diseases. In leishmaniasis, IVM displays notable antileishmanial activity, showcasing prophylactic effects and reduced parasite load (Freitas et al., 2023; Hanafi et al., 2011; Reis et al., 2021). For trypanosomiasis, IVM demonstrates efficacy against various stages of *Trypanosoma* parasites, presenting both trypanostatic and trypanocidal effects, and it also holds potential for controlling vectors (Fraccaroli et al., 2022; Gupta et al., 2022; Katsuno et al., 2015; Mosquillo et al., 2018; Pooda et al., 2013; Udensi and Fagbenro-Beyioku, 2012). In the case of Malaria, IVM exhibits capacity as an antimalarial agent, targeting multiple species and stages of the parasite, with added mosquitocidal and larvicidal activities (Foley et al., 2000; Kobylinski et al., 2012; Kobylinski et al., 2017). Its consideration in schistosomiasis treatment, alongside praziquantel, highlights its effectiveness in reducing worm and egg burdens, though further research is essential (Katz et al., 2017; Vicente et al., 2021). In trichinosis, IVM proves effective against mature larvae, showcasing versatility across different life-cycle stages (Elmehy et al., 2021; Mukaratirwa et al., 2008; Soliman et al., 2011). Overall, IVM’s diverse applications underscore its significance as a promising therapeutic agent against various parasitic diseases, signaling a comprehensive approach to global health challenges.

In *G. lamblia*, in concentration tests ranging from 25 μM to 100 μM, it was found that 60 μM and 80 μM of IVM slightly inhibit parasite growth (Mayol et al., 2020). This study aims to elucidate the mechanism of cell death exerted by IVM during parasite growth, highlighting its potential as a novel anti-giardial agent. The investigation focuses on regulated necrosis or apoptosis, two distinct types of cell death. Necrosis involves passive cell death due to irreversible damage, whereas apoptosis is a metabolic process involving mitochondria (Henry et al., 2013; Ledda-Columbano et al., 1991; Taylor et al., 2008). IVM likely employs alternative mechanisms in *G. lamblia* since this parasite lacks mitochondria and a conventional endosomal-lysosomal system. In this study, this drug demonstrated significant cytotoxicity against *G. lamblia* trophozoites, particularly at higher concentrations. Determination of the half-maximal inhibitory concentration (IC_50_) revealed a dose-dependent effect, hindering trophozoite growth. IVM treatment led to elevated ROS levels in the treated cells, with a concentration-dependent increase observed in both the cytoplasm and nuclei of trophozoites. Additionally, exposure to IVM resulted in DNA fragmentation. Annexin V and PI (Propidium Iodide) staining revealed higher levels of cell death through apoptosis and necrosis in trophozoites treated with higher drug concentrations. Our findings revealed that the treatment caused significant arrest in the S phase of the cell cycle in *G. lamblia* trophozoites and induced structural changes. These findings highlight the drug’s mechanism of action and its potential as a promising anti-giardial agent and contribute to developing enhanced therapeutic approaches for giardiasis.

## Materials and methods

### Organisms and *in vitro* cultures

The trophozoites of *G. lamblia* used in this study were obtained from the WB strain clone 1267, available at the American Type Culture Collection (ATCC 50582) (Gillin et al., 1990). Axenic cultures of trophozoites were maintained in TYI-S-33 medium supplemented with 10% v/v adult bovine serum and 5% w/v bovine bile (Gillin and Diamond, 1981). The cultures were performed in borosilicate tubes with screw caps (Eurotubo Deltalab). To collect microorganisms, tubes containing actively growing cells were chilled at 4 °C for 30 min and then harvested by centrifugation at 2500 rpm for 15 min at 4 °C.

### Ivermectin treatment

IVM, acquired from Sigma-Aldrich (St. Louis, MO, USA), was dissolved in dimethyl sulfoxide (DMSO) to its maximum solubility, resulting in a stock solution with a 56 mg/mL concentration. Parasites (5 × 10^5^ cells) were cultured for 24 h in IVM, whereas the control group was treated with 0.5% v/v DMSO (vehicle).

### Cell proliferation assay

The MTT ((3-(4, 5-dimethylthiazol-2-yl)-2,5-diphenyl tetrazolium bromide)) colorimetric assay was performed to investigate the cytotoxic potential of IVM (Joray et al., 2015; Mosmann, 1983). In summary, 5 × 10^5^ trophozoites suspended in 100 μL of growth medium were seeded in 96-well plates from Greiner Bio-One (Germany), containing 100 μL of complete medium and serial two-fold dilutions of IVM dissolved in 0.5% v/v DMSO, a concentration at which no adverse effects on cell growth were observed. The drug was evaluated at the final maximum concentration of 100 μM. After 48 h of anaerobic incubation in plates in a CO_2_ chamber, the plates were washed three times with sterile PBS by centrifugation. The trophozoites were suspended in 200 μL of PBS. Then, 20 μL of MTT solution (5 mg/mL in sterile PBS, pH 7.4) was added, and the mixture was incubated for 4 hours. After incubation, it was centrifuged at 2000 rpm for 5 minutes. The supernatants were discarded, and the formazan crystals produced by metabolically active cells were dissolved in 100 μL of DMSO. The absorbance was measured at 595 nm using a microplate reader. Two wells duplicates were used for each concentration of IVM, and three independent experiments were conducted. Untreated and DMSO-treated cells (0.5%) were used as controls. The percentage of cytotoxic activity of IVM was determined using the following formula: cytotoxicity (%) = [1 - (optical density of treated cells - optical density of DMSO)/(optical density of control cells treated with DMSO - optical density of DMSO)] × 100 (Joray et al., 2015). The obtained values were used to calculate the IC_50_ of the compound through non-linear regression using GraphPad Prism 9.0 software (GraphPad Software, Inc., CA, USA), following the method described by (Mosmann, 1983). The results are expressed as the mean ± SE.

### Detection of oxidative DNA damage

Reactive oxygen species (ROS) formation was evaluated using the fluorescent probe 2′,7′-dichlorodihydrofluorescein diacetate (H2DCFDA). Trophozoites were exposed to IVM at concentrations of 40, 60, and 80 μM, washed with PBS, and incubated for 30 min at 37 °C with a fluorescent tracer at 25 μM (Image-ITTM Live Green ROS Detection kit; Life Technologies, USA). Subsequently, Hoechst 33342 was added at a final concentration of 1 μM for 5 minutes. Finally, the fluorescence was visualized using an FV1200 confocal microscope (Center for Micro and Nanoscopy of Córdoba. CEMINCO).

### TUNEL assay

Treated and untreated cells were detached by incubation in an ice-water bath for 30 minutes, harvested by centrifugation at 2500 rpm for 10 minutes at 4 °C, and washed twice with PBS. Parasites were resuspended in 1 mL of PBS medium 1% v/v and a drop were placed in coverslips pre-treated with polyLysine. Then, coverslips were incubated at 37 °C for 30 min to allow the trophozoites to adhere. The cells were fixed in 4% v/v formaldehyde in PBS for 40 min, then washed two times with PBS for 5 min, permeabilized with 0.1% v/v Triton X-100 for 30 min, and washed two times with PBS for 5 min. Finally, the TUNEL (Terminal deoxynucleotidyl transferase dUTP Nick-End Labeling) assay was carried out using a Situ Cell Death Detection Kit, TMR red (TUNEL technology, Roche), according to the manufacturer’s protocol.

### Annexin V/PI dual staining assay in trophozoites

Annexin V and PI staining were performed using a commercial kit (Dead Cell Apoptosis Kit with Annexin V Alexa Fluor488 & Propidium Iodide, Invitrogen™) following the manufacturer’s protocol. Briefly, treated and untreated trophozoites were washed with cold PBS medium (1%), and the cell pellet was resuspended in 1X Annexin binding buffer and kept on ice. Next, 5 μL of Annexin V Alexa Fluor^®^ 488 (which binds to phosphatidylserine exposed on the outer leaflet of the plasma membrane, an early marker of apoptosis) and 1 μL of a working solution of PI (which penetrates cells with compromised membranes, indicating late apoptosis or necrosis) were added. The cells were then incubated at room temperature in the dark for 15 min. Data were collected using a BD FACSCanto™ (BD Biosciences) at the CIBICI-CONICET-UNC and analyzed by flow cytometry. Annexin V-positive and PI-negative cells were considered apoptotic (early apoptosis), while cells positive for both Annexin V and PI were classified as in late apoptosis or necrotic. Cells positive for PI but negative for Annexin V were considered necrotic. Results were expressed as mean ± SD. Data were analyzed using one-way analysis of variance (ANOVA) with Tukey’s *post hoc* test. GraphPad Prism 9.0 was used, with p ≤ 0.05 considered statistically significant. Additionally, fluorescence was examined using an FV1200 confocal microscope (Center for Micro and Nanoscopy of Córdoba. CEMINCO). The experiments were conducted in triplicate.

### Cell cycle distribution by flow cytometry

Two million cells in the exponential growth phase were utilized in each culture set, including the negative control (cells treated only with the vehicle, DMSO) and IVM-treated cells at 40, 60, and 80 μM. Following treatment, cells were harvested and washed twice with ice-cold PBS. Subsequently, they were fixed in 70% v/v cold ethanol and stored at 4 °C for 48 h. Before analysis, cells were washed twice with PBS and stained with a solution containing propidium iodide (PI, 2 μg/mL, Sigma-Aldrich CO, USA) and RNase A (50 μg/mL, Sigma-Aldrich CO, USA) in PBS (pH 7.4) overnight at 4 °C in the dark. DNA content was assessed using flow cytometry with the BD FACSCanto™ (BD Biosciences) located at CIBICI-CONICET-UNC. The relative distribution of at least 20,000 cells was analyzed using the FlowJo software version 7.6.2 (Tree Star, Inc. OR, USA).

### Ultrastructural analysis by transmission electron microscopy (TEM)

After the treatments, control or treated trophozoites were washed with PBS and fixed in a Karnovsky mixture containing 4% (w/v) formaldehyde and 2% (w/v) glutaraldehyde in 0.1 M cacodylate buffer for 2 h. Then, fixed cells were centrifuged, and the pellets were washed and treated with 1% OsO_4_ for 1 h. Cells were embedded in Spur resin after dehydration with a series of graded cold acetones. Following a 48-h polymerization at 60 °C, 90 nm ultrathin sections were cut using an RMC Power Tome -XL ultramicrotome with a diamond knife. Finally, the grids were examined using a Hitachi HT 7800 electron microscope (Hitachi, Tokyo, Japan) operated at 80 kv and photographed with an NS 15 AMT camera (Advanced Microscopy Techniques Corp., Woburn, MA, USA) located at Electron Microscopy Center, Faculty of Medical Sciences, UNC.

### Immunofluorescence assay (IFA) and confocal microscopy

The IFA of fixed cells was performed as described by Rivero et al. (2010). Briefly, trophozoites were cultured in a growth medium, harvested, and attached to slides pre-treated with polyLysine. After fixation with 4% v/v formaldehyde, the cells were blocked with 3% g/v bovine serum albumin (BSA) in PBS Tween 0.05% v/v. The cells were then incubated with the anti-alpha tubulin (Sigma, St. Louis, MO) or anti-alpha acetylated tubulin (Sigma) in 1.5% g/v BSA in PBS Tween 0.05% v/v, followed by incubation Goat anti-Mouse IgG Secondary Antibody™, Alexa Fluor 488 (ThermoFisher Scientific) in 1.5% g/v BSA in PBS Tween 0.05% v/v. The preparations were washed with PBS and mounted in FluorSave™ mounting medium. Fluorescence staining was visualized with a motorized FV1200 Olympus confocal microscope (Olympus UK Ltd, UK), with 63X oil immersion objectives (Numerical Aperture 1.32). Differential interference contrast (DIC) images were collected simultaneously with the fluorescence images using a transmitted light detector. Images were processed using Fiji software (Schindelin et al., 2012).

## Results

### Ivermectin exhibits cytotoxicity against *Giardia lamblia* trophozoites

**FIGURE 1:**
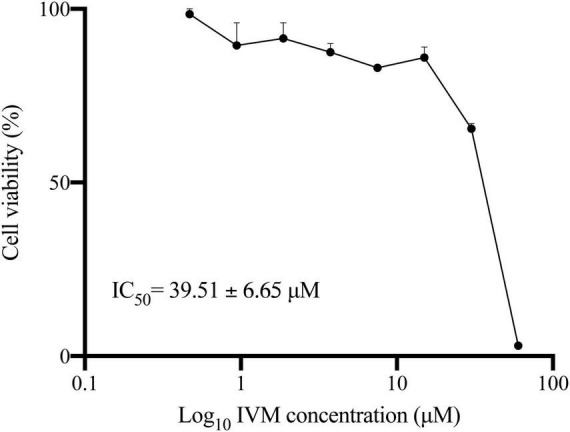
Dose-Response Curves Showing the Relationship Between *Giardia lamblia* Trophozoites’ Viability and Log_10_ Ivermectin (IVM) Concentration. Viability of trophozoites 48 hours post-treatment with increasing concentrations of IVM was assessed using the MTT assay. Values represent mean ± standard error (SE) from at least three independent experiments.

Our previous investigations showed that Ivermectin (IVM) demonstrates cytotoxicity against *Giardia lamblia* trophozoites at high concentrations (Mayol et al., 2020). Here, we determined the half-maximal inhibitory concentration (IC_50_) of IVM on *G. lamblia* trophozoites by determining the percentage of cell viability relative to the Log_10_ of IVM concentration (expressed in μM) (Figure 1). The results showed a decrease in cell viability as the drug concentration (0.47 to 60 μM) increases, with an IC_50_ value of 39.51 ± 6.65 μM. Further analysis indicated that IVM exhibited high cytotoxic effects on *G. lamblia* trophozoite, inducing 85.25 ± 3.42% and 97.25 ± 1.54% cell death at 60 μM and 80 μM concentrations, respectively. Thus, to investigate whether IVM induces apoptosis or necrosis, we decided to perform the next experiments by subjecting *G. lamblia* trophozoites to a 24-hour treatment period using three different concentrations of IVM (40 μM, 60 μM, and 80 μM). This approach allowed us to examine the cell death mechanisms of IVM in still-alive cells.

### Ivermectin elicits ROS production

**FIGURE 2:**
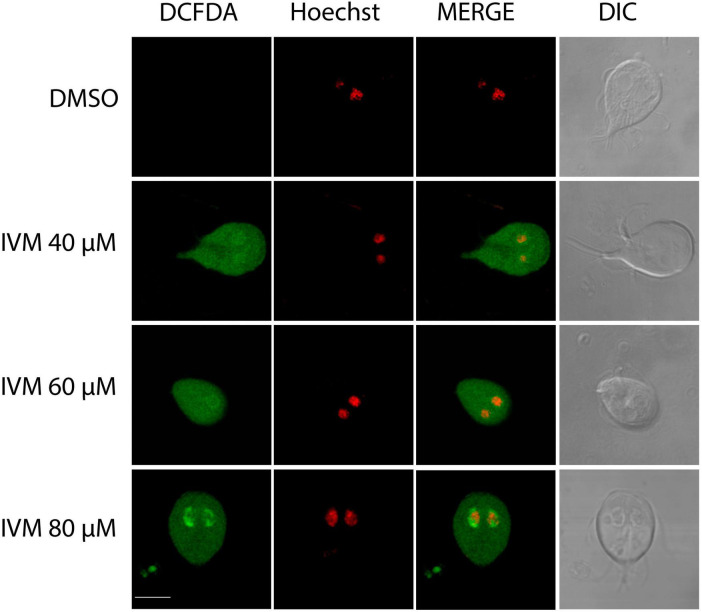
Reactive Oxygen Species (ROS) Production in *Giardia lamblia* Trophozoites Exposed to Ivermectin. Trophozoites were exposed to vehicle (DMSO) and 40 μM, 60 μM, or 80 μM IVM for 24 hours at 37 °C. ROS production was measured using DCFDH. Metronidazole served as a control for intracellular ROS production. Micrographs depict trophozoites stained with DCFDH (left panels), nuclei stained with Hoechst (middle panels), and Differential Interference Contrast microscopy (DIC) (right panels). Increased ROS production was observed after IVM treatment compared to untreated cells. Images are representative of three independent experiments. Scale bars = 5 μm.

Oxidative stress involves the increase of reactive oxygen species (ROS) within cells, which can damage lipids, proteins, and DNA (Kho et al., 2018). Interestingly, in several cancer cell model studies, oxidative stress has been linked to IVM, demonstrating a significant increase in intracellular ROS (Liu et al., 2016). We treated parasites with IVM at 40, 60, and 80 μM concentrations, analyzing at least 100 cells for each condition using confocal microscopy. Compared to untreated controls, higher ROS levels were observed in cells treated with 40, 60, and 80 μM IVM. Confocal microscopy analysis revealed fluorescent staining indicative of ROS formation in most of the treated cells (Figure 2). Detailed analysis of individual cells showed a widespread distribution of ROS throughout the trophozoite cytoplasm during incubation with the different doses of IVM (Figure 2). Additionally, a dose-dependent increase in ROS staining within the nuclei of trophozoites was observed (Figure 2). These findings indicate that IVM triggers both cytoplasmic and nuclear ROS signaling in a concentration-dependent manner, suggesting it has complex oxidative stress effects on the parasite.

### Ivermectin induces DNA fragmentation

**FIGURE 3:**
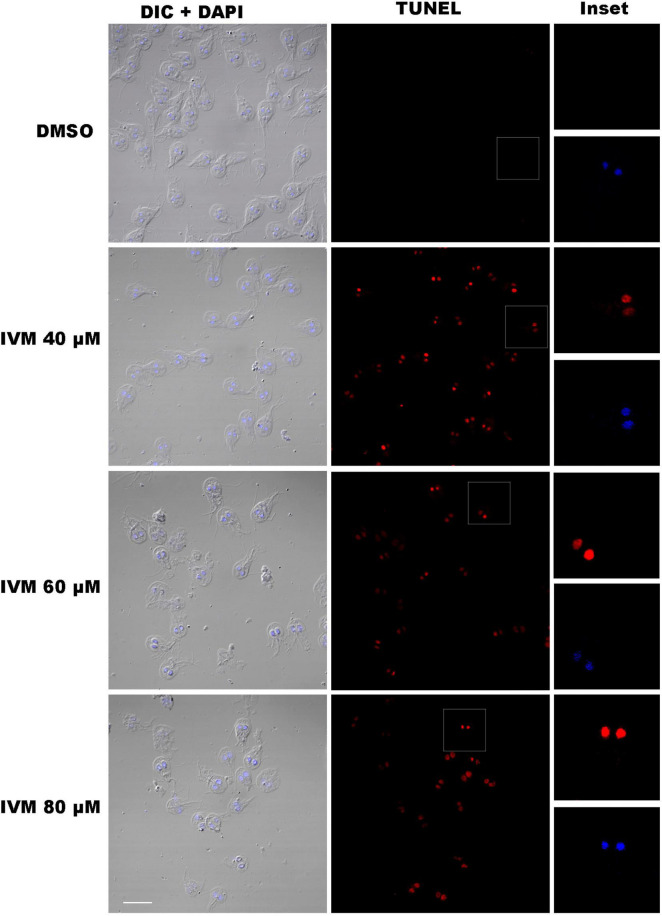
DNA Fragmentation in *Giardia lamblia* Trophozoites Treated with Ivermectin. TUNEL assays were performed on trophozoites treated with 40 μM, 60 μM, and 80 μM ivermectin (IVM), and DMSO (vehicle control). The first column shows DIC + DAPI-stained cells, the second column shows TUNEL-positive cells (red), and the third column provides inset images highlighting TUNEL and DAPI signals. Trophozoites treated with IVM display high levels of DNA fragmentation, visible as red fluorescence in the TUNEL channel, in contrast to the control (DMSO). Scale bars = 10 μm.

Oxidative damage from intracellular ROS can cause DNA damage, including base modifications and single- and double-strand breaks, among other lesions that are often toxic and mutagenic (Cheung et al., 2024). A TUNEL assay was performed to investigate further DNA fragmentation in *G. lamblia* trophozoites exposed to IVM. TUNEL-positive cells, indicating DNA strand breaks, were significantly more prevalent in trophozoites treated with the drug than in untreated controls. No difference in fluorescence intensity was evident between IVM 40, 60, and 80 μM (Figure 3). The DNA damage signal was detected in the nucleus, as the TMR red signal overlapped with that of DAPI. In contrast, the control cells displayed no fluorescence, indicating no endogenous DNA fragmentation. These results suggest that IVM induces substantial DNA damage in G. *lamblia*, as confirmed by the TUNEL assay, further supporting its cytotoxic effects on the parasite.

### Ivermectin triggers cell death in a concentration-dependent manner

**FIGURE 4:**
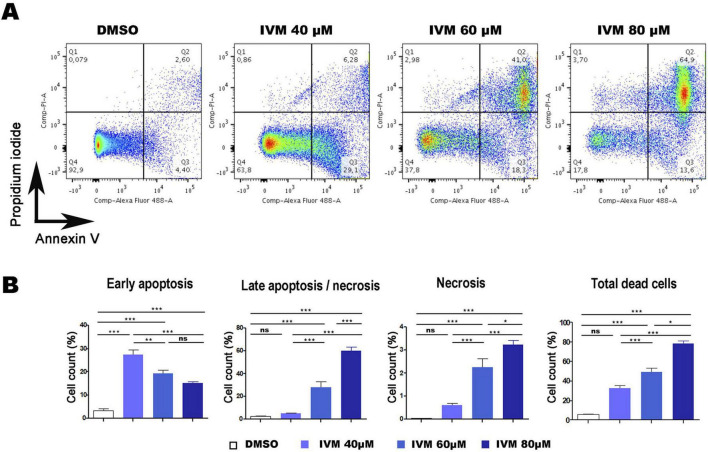
Ivermectin-Induced Cell Death in *Giardia lamblia* Trophozoites Analyzed by Flow Cytometry. **(A)** Dot plots illustrating cell death in trophozoites treated with 0.5% DMSO (negative control) or 40 μM, 60 μM, or 80 μM IVM for 24 hours, stained with Annexin V-Alexa Fluor^®^ 488 and PI. **(B)** Graphs showing percentages of cells in early apoptosis, late apoptosis/necrosis, necrosis, and total dead cells (lower right). Data represent mean ± SD of three independent experiments. *p < 0.05, **p < 0.01, ***p < 0.001, ANOVA followed by Tukey’s Multiple Comparison Test.

Flow cytometry and confocal microscopy were used to investigate the programmed cell death mechanism with the commercial Dead Cell Apoptosis Kit containing Annexin V Alexa Fluor 488 and Propidium Iodide (PI). One indicative feature of apoptosis is the externalization of phosphatidylserine (PS), detected by Annexin V on the plasma membrane. DNA degradation, a key cell death marker, was evaluated using PI, a membrane-impermeable nucleic acid-specific dye. Flow cytometry allowed the analysis of cells in early apoptosis (Q3), late apoptosis/necrosis (Q2), necrosis (Q1), and the total percentage of dead cells (Q3 + Q2 + Q1). Dot plots revealed a concentration-dependent increase in cell death (Figure 4A). Trophozoites treated with 0.5% DMSO (vehicle control) exhibited 99.2% cell viability (Q4). In contrast, incubation with 40 μM, 60 μM, and 80 μM IVM resulted in viabilities of approximately 63.8%, 37.8%, and 17.8%, respectively.

**FIGURE 5:**
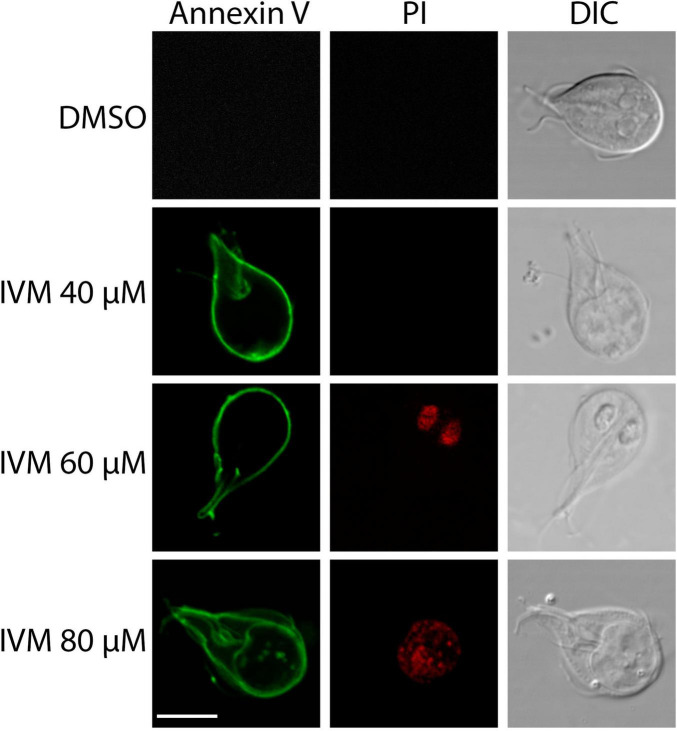
Analysis of programmed cell death in trophozoites treated with IVM. Fluorescence confocal microscopy images showing Annexin V (green) and PI (red) staining in untreated trophozoites (Control) and those treated with 40, 60, or 80 μM IVM for 24 hours. DIC: Differential Interference Contrast. Scale bars = 5 μm.

Dots Plots illustrated that at 40 μM IVM, around 30% of cells were in early apoptosis (Annexin V + /PI-), 6.28% in late apoptosis (Annexin V + /PI +), and 0.86% in necrosis (Annexin V-/PI +). At 60 μM IVM, early apoptosis decreased to 18.3%, while late apoptosis increased to 41% and necrosis to 2.98%. With 80 μM IVM, the percentages were 13.6% in early apoptosis, 64.9% in late apoptosis, and 3.70% in necrosis (Figure 4A). The statistical analysis obtained is shown in Figure 4B. When the trophozoites were analyzed by confocal microscopy, we observed no staining for control cells with vehicle while increasing exposure to IVM resulted in green staining of Annexin V. At least 100 cells from each condition were evaluated (Figure 5). Only at 60 and 80 μM of IVM treatment, red nuclear staining with PI was observed (Figure 5). Note that at 60 and 80 μM of IVM, a significant increase in nuclear DNA degradation was observed, as evidenced by the dispersion of the red PI marker (Figure 5). In summary, ivermectin-induced cell death is dose-dependent, with lower concentrations primarily inducing apoptosis and higher concentrations leading to necrosis.

### Ivermectin induces cell cycle arrest in S phase

**FIGURE 6:**
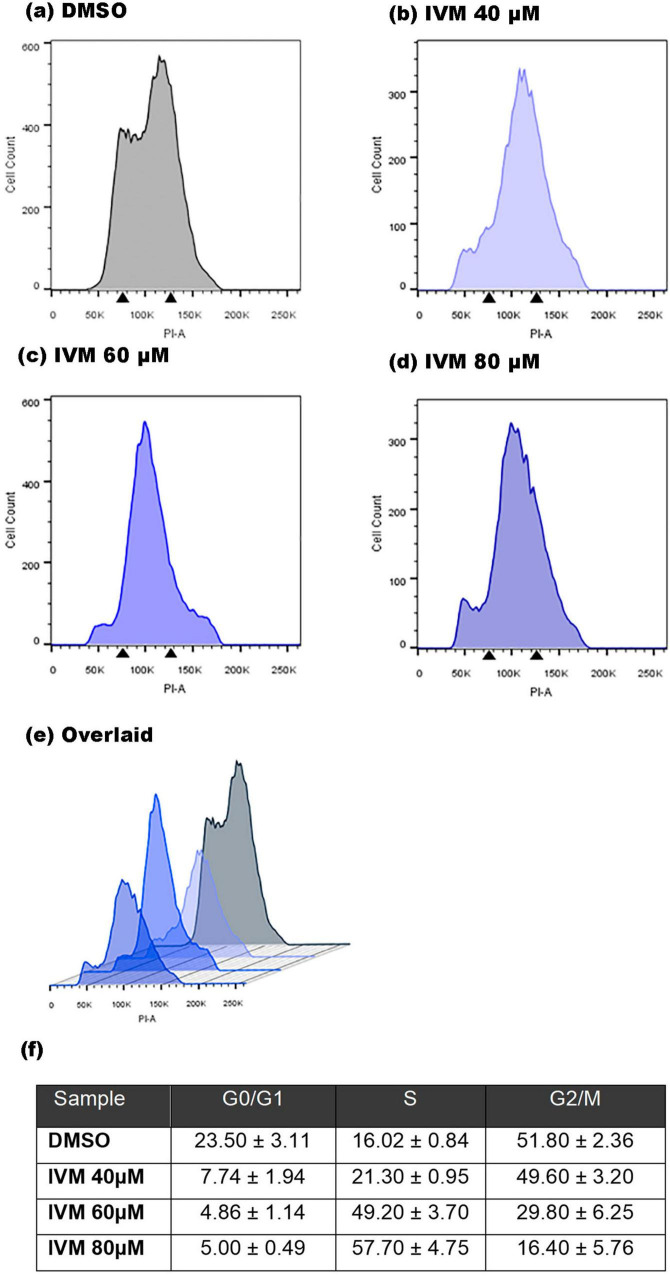
Cell cycle analysis of *Giardia lamblia* treated with IVM by flow cytometry. Histograms depict trophozoites incubated for 24 hours with **(a)** vehicle (DMSO), **(b)** 40 μM IVM, **(c)** 60 μM IVM, and **(d)** 80 μM IVM, with **(e)** an overlay of all treatments including the control. The percentages of cells in each cell cycle phase are provided **(f)**. After drug exposure, trophozoites were fixed, stained with PI in the presence of RNase, and analyzed by flow cytometry. Arrows indicate G1 and G2/M phases. PI-A corresponds to PI staining intensity. Results are mean ± SD of three independent experiments.

Investigating the cell cycle dynamics following IVM treatment in *G. lamblia* is crucial for understanding the drug’s mechanisms of action. Thus, the nuclei of the trophozoites were stained with PI to determine whether IVM treatment can affect the progression of the cell cycle. Following 24 hours of drug treatment, the percentage of trophozoites in the G0/G1 phase showed an apparent decline, while the S phase experienced a significant surge (Figures 6a–f). Notably, the percentage of cells in the G2/M phase showed a marked decrease in parasites treated with various concentrations of IVM, underscoring the contrasting effects of IVM compared to the control group. The S phase of the cell cycle is when DNA replication occurs. This arrest in the S phase could interfere with DNA replication, potentially preventing cells from dividing and multiplying.

### Ivermectin causes ultrastructural damage

**FIGURE 7:**
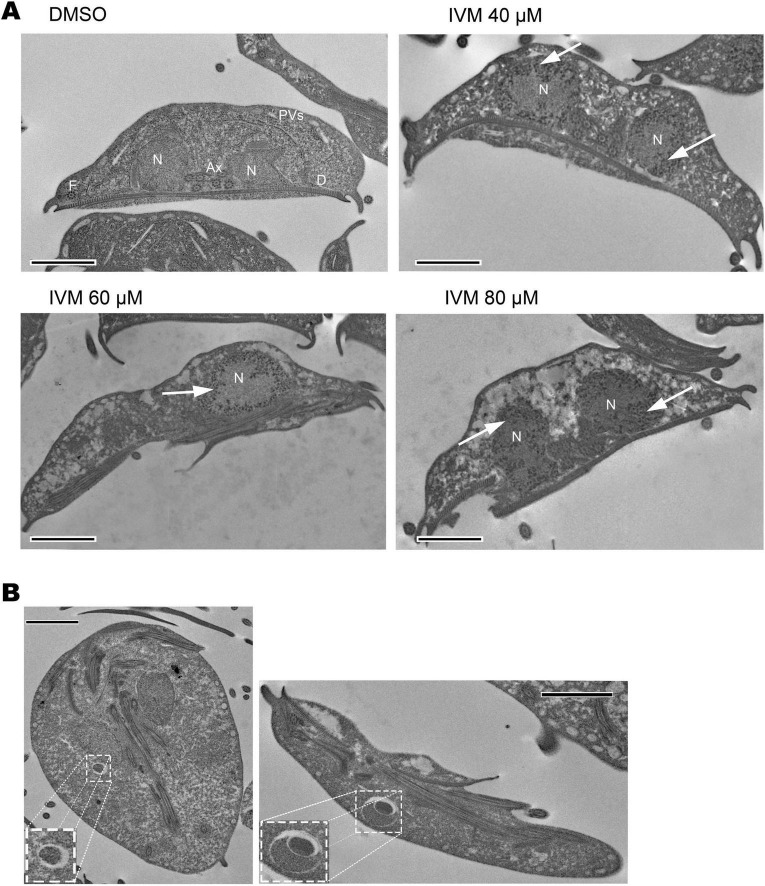
Analysis of ultrastructural damage in trophozoites treated with Ivermectin, detected by electron microscopy. Structural features including nuclei (N), ventral disc (D), peripheral vesicles (PVs), axonemes (Axs), and flagella (F) are shown. **(A)** Trophozoites incubated for 24 hours with vehicle (DMSO), IVM at 40 μM, 60 μM, and 80 μM concentrations. Cytoplasmic vacuolization and chromatin condensation are evident post-treatment. **(B)** Trophozoites treated with 80 μM IVM exhibit unusual intracellular structures (enlarged view). Scale bars: 2 μm.

The parasites were analyzed after exposure to IVM for 24 h, and the effects of the drug on the trophozoite structure were observed. While typical cell structures such as nuclei, peripheral vacuoles, axonemes, ventral disk, and glycogen granules were uniformly distributed throughout the cytoplasm in control samples (Figure 7A), significant alterations were observed in trophozoites exposed to IVM. These included cytoplasmic vacuolization and pronounced chromatin condensation (distributed in the nucleus periphery) all indicative of an apoptotic process, with these effects becoming more pronounced as the concentration of IVM increased (Figure 7A). Although morphological changes occurred, the general shape of the parasite was preserved after administration of the highest concentrations of the compound (Figure 7A). Furthermore, other cell structures such as the ventral disk and flagella were not altered after IVM treatment. An important observation in trophozoites treated with 80 μM of IVM was the emergence of vacuoles ranging from 500 nm to 1 micrometer (Figure 7B). These vacuoles were only observed at higher concentrations of IVM (Figure 7B). All observed changes align with an apoptotic-like process: trophozoites exhibiting cytoplasmic vacuolization and chromatin condensation, with effects intensifying at higher drug concentrations.

### High concentrations of ivermectin lead to alterations in α-tubulin distribution in trophozoites

**FIGURE 8:**
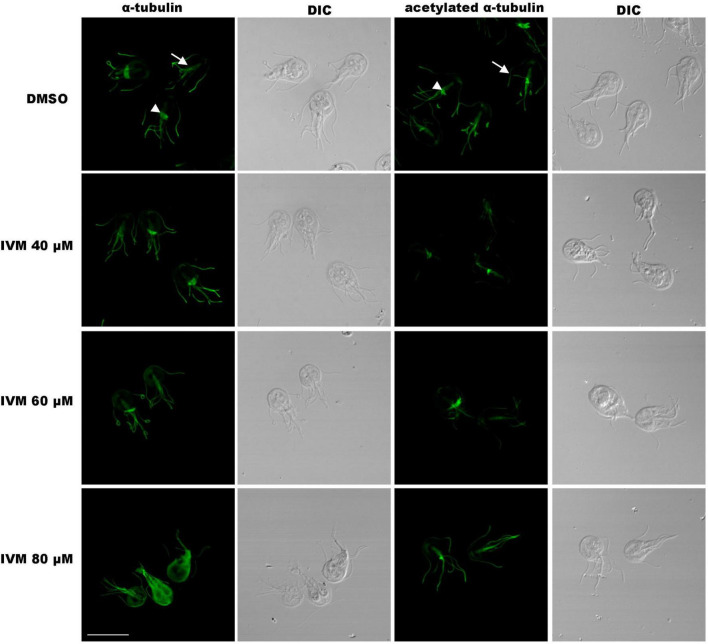
Immunofluorescence microscopy of trophozoites after IVM treatment. Cells stained with anti-α-tubulin or anti-acetylated α-tubulin monoclonal antibodies. In vehicle (DMSO) controls, positive labeling of α-tubulin and acetylated α-tubulin is observed in the median body (MB), flagella (F), and ventral disk (VD). Treatment with lower IVM concentrations (40, 60 μM, 24 h) shows no alteration in tubulin distribution. However, incubation with 80 μM IVM for 24 h leads to redistribution of total tubulin to the cytosol (asterisk), while acetylated tubulin decreases in the median body (arrow). DIC: Differential Interference Contrast. Scale bar = 10 μm.

Although TEM did not reveal structural changes in the ventral disc and flagella after IVM treatment, studying the localization of α-tubulin, a key cytoskeleton component, can provide insights into the structural integrity of the trophozoites under drug treatment. Therefore, we considered it essential to investigate its localization after incubation with IVM. IFA and confocal microscopy using an anti-α-tubulin monoclonal antibody, showed pear-shaped, binucleated trophozoites with typical α-tubulin distribution in the median body (arrowhead), flagella (head), and ventral disk (Figure 8). However, after treatment with 80 μM IVM, a redistribution of α-tubulin towards the cytosol was identified, accompanied by the disappearance of localization in the median body (Figure 8). IFA using an anti-acetylated tubulin monoclonal antibody revealed no changes between the control cells and those treated with 40 and 60 μM of IVM (Figure 8A). However, after treatment with 80 μM IVM, the marking in the median body tends to diminish. These findings underscore the concentration-dependent effects of IVM on α-tubulin distribution in *G. lamblia* trophozoites.

## Discussion

This study investigates the cytotoxic effects of ivermectin (IVM) on *Giardia lamblia* trophozoites. Our findings reveal dose-dependent cytotoxicity, induction of cell death pathways, increased reactive oxygen species (ROS), and DNA fragmentation, causing trophozoite arrest in the cell cycle’s S phase.

The efficacy of IVM in *Giardia* infection was evaluated in a rat model (Foletto et al., 2015). Ivermectin was administered orally in water at a dose of 48 mg/L (resulting in a calculated blood and intestinal concentration of approximately 54.9 μM) and was shown to be effective in treating *Giardia* spp. infection in laboratory rats, supporting the safety of IVM use at similar concentrations. However, naturally infected rats are not considered ideal models for studying *Giardia* infections in humans, particularly for *Giardia lamblia*, as rats are susceptible to *Giardia muris*, a species not closely related to *G. lamblia*. Gerbils are often used as an alternative rodent model for *G. lamblia* studies, as they are more susceptible to human-infective strains (Assemblages A and B) and exhibit pathologies more like those seen in humans. Compared to our previous results, where lethality was observed only at 100 μM (Mayol et al., 2020), our current study found significant cytotoxicity of IVM against *G. lamblia* trophozoites at 60 μM and 80 μM. This discrepancy may be attributed to the more sensitive MTT method we used for assessing cytotoxicity, as opposed to the trypan blue and Neubauer chamber method used in the earlier study. Regarding the doses used in this study, although recent research indicates that intestinal Caco-2 cells are sensitive to IVM at around 10 μM, similar to other mammalian cells (Houshaymi et al., 2019), *in vitro* cytotoxicity assays may not accurately reflect *in vivo* conditions, where factors like drug absorption, metabolism, and microbiota interactions are significant. Future studies in more complex biological models, such as the gerbil model, must fully assess IVM’s potential for treating giardiasis.

*Giardia* and other protozoan parasites, including *Plasmodium*, *Entamoeba histolytica*, *Trypanosoma*, and *Leishmania*, thrive in environments with low oxygen tension, a condition referred to as microaerophilic (Kho et al., 2018). Specifically, *G. lamblia* can only endure oxygen concentrations ranging from 0 to 50 μM (Lloyd et al., 2000), making it highly susceptible to oxygen and ROS. This susceptibility is attributed to the absence of conventional ROS-scavenging enzymes (CAT, SOD, GPx) and the presence of ROS-generating enzymes like NAD(P)H: menadione oxidoreductase. Additionally, the parasite possesses vital metabolic enzymes (pyruvate-ferredoxin oxidoreductase) sensitive to oxygen (Li and Wang, 2006; Mastronicola et al., 2014). In various investigations on mammalian cells, the administration of IVM has been associated with the induction of oxidative stress (Fan et al., 2024). Similarly, research on *Leishmania* has revealed that IVM triggers mitochondrial membrane potential depolarization and elevates ROS levels, accompanied by the formation of lipid bodies, suggesting the establishment of a stress environment leading to *Leishmania* death (Reis et al., 2021). In this context, our study aimed to evaluate IVM’s potential to induce oxidative stress in the protozoan parasite *G. lamblia*. The results indicated that exposure to IVM resulted in the generation of intracellular ROS in trophozoites susceptible to the drug. ROS was predominantly localized in the nuclei, and their labeling intensity correlated with the drug concentration. This observation aligns with previous reports where trophozoite nuclei were identified as the primary site of ROS formation following treatment with albendazole concentrations of 1.35 μM and 8 μM (Martinez-Espinosa et al., 2015). Thus, the cytotoxic impact of IVM on this protozoan parasite may involve oxidative stress. Future research should focus on identifying the specific ROS implicated and elucidating the mechanisms responsible for their formation.

ROS can cause permanent damage to various cellular components through the oxidation of proteins, carbohydrates, lipids, and nucleic acids, followed by membrane modification, receptor alteration, cytoskeletal protein inactivation, enzyme inactivation, and genome damage (Chiumiento and Bruschi, 2009). In our study, the TUNEL assay confirmed significant DNA fragmentation in *G. lamblia* trophozoites treated with IVM at 40, 60, and 80 μM concentrations, highlighting DNA as a critical target of IVM action. Similar DNA damage has been reported in *Giardia* following treatment with drugs such as albendazole (Martinez-Espinosa et al., 2015) and metronidazole (Ghosh et al., 2009). A previous study in *Giardia* suggested that DNA damage results from the formation of 8-hydroxy-2′-deoxyguanosine (8OHdG) adducts due to the oxidative environment induced by metronidazole (Ordonez-Quiroz et al., 2018). Similarly, our work demonstrated precise detection of DNA strand breaks in individual cells after incubation with IVM at concentrations of 40, 60, and 80 μM. This fragmentation aligns with an apoptosis-like process previously observed in *Giardia* under oxidative stress and metronidazole treatment (Bagchi et al., 2012). Indeed, detecting phosphatidylserine translocation to the outer side of the cell membrane suggested apoptosis. Our results support a process of early apoptosis at concentrations near 40 μM and late apoptosis/necrosis at higher concentrations of IVM. *G. lamblia* is of interest to parasitologists and evolutionary biologists due to its strategic phylogenetic position, which allows for hypotheses about early events in eukaryotic evolution. A distinctive feature is its evolutionary divergence, lacking caspases and Bcl-2-like proteins-integral components of apoptosis (Ghosh et al., 2009). Furthermore, it lacks mitochondria, where oxidative phosphorylation occurs and is a critical process in cellular death pathways (Adam, 2021). Our results suggest that IVM could trigger a process resembling apoptosis in *G. lamblia*, despite differences in conventional pathways and the absence of mitochondria and characteristic proteins previously described in other eukaryotic organisms.

Interestingly, trophozoites exposed to increasing concentrations of IVM for 24 hours experienced a partial arrest in the S phase of the cell cycle, showing oxidative stress, DNA degradation, and an apoptosis-like process. Although this arrest was not complete, allowing some cells to continue through the cycle, a dose-dependent effect was observed, similar to findings in bovine mammary epithelial cells (Park et al., 2020). These observations align with previous results from our laboratory regarding IVM’s impact on *G. lamblia* reproduction and growth (Mayol et al., 2020). The S phase arrest may trigger an apoptotic pathway akin to what was observed in *G. lamblia* treated with Kaempferol, where a cytostatic effect was attributed to impaired DNA replication and halted cytokinesis (Argüello-García et al., 2020). In many cases, DNA damage leads to S phase arrest as a protective mechanism, allowing time for repair. However, prolonged arrest can exacerbate DNA damage, mainly if the replication machinery is stalled. We hypothesize that ROS generated by IVM likely caused this DNA damage, which induced cell cycle arrest to prevent replication of compromised DNA and facilitate repair. Supporting IVM’s role in cell cycle arrest, our group demonstrated that this drug also inhibits Importin-α/Importin-β-mediated nuclear transport, disrupting the encystation process by arresting *Giardia* in the trophozoite stage and preventing cyst formation(Mayol et al., 2020).

To thoroughly understand the effects of IVM treatment at the cellular level, we conducted detailed morphological assessments using TEM analysis. Remarkably, upon drug administration, cells displayed significant vacuolation. We also observed notable changes in nuclear structure, characterized by chromatin condensation. These compacted areas of chromatin result from DNA fragmentation. This process has been described in various circumstances, such as during apoptosis or in response to specific treatments or stimuli that affect the structure and organization of DNA in the cell nucleus (Benchimol et al., 2023; Gadelha et al., 2019). Additionally, the presence of unusual intracellular structures following treatment with the highest IVM concentration potentially corresponds to autophagosomes. However, the characteristic double concentric membrane of these vacuoles is not observed, thus, a more detailed study with specific markers would be necessary to define these atypical structures found.

The structure and functions of the median body remain subjects of debate are still under debate (Hagen et al., 2020). It comprises semi-organized microtubules (MTs) formed by tubulin and beta-giardin proteins (Crossley et al., 1986) and is dynamic during interphase, being sensitive to both MT-stabilizing and MT-depolymerizing drugs (Dawson et al., 2007; Sagolla et al., 2006). The median body’s shape and presence varies during the cell cycle; it disappears altogether following mitosis, just before disc division (Sagolla et al., 2006). In our study, the cell cycle arrest in the S phase caused by IVM likely disrupts median body assembly, as its dynamics are tightly linked to cell cycle progression. This disruption could impair its function as a tubulin reservoir, affecting the duplication of MT structures necessary for *Giardia*’s attachment and division. We suggest that the increased tubulin signal observed after IVM treatment reflects not an upregulation of protein levels, but rather a redistribution of tubulin from basal bodies, which act as reservoirs. This redistribution may support microtubule reorganization or repair processes triggered by drug-induced stress.

Ivermectin’s mechanism in *Giardia* (Mayol et al., 2020) differs from its known action in invertebrates (Liu et al., 2020; Sulik et al., 2023) as *Giardia* lacks the glutamate-gated chloride channels that ivermectin typically targets (Adam, 2021). Instead, in the present study, we demonstrated that IVM induces ROS, which likely play a crucial role in its cytotoxic effect. Elevated ROS levels lead to oxidative damage, membrane disruption, and DNA breaks, ultimately resulting in apoptosis-like cell death. Further studies are needed to clarify the molecular mechanisms behind IVM-induced cell cycle arrest, its effect on median body assembly and function, and to explore potential synergistic effects with other therapeutic agents. Understanding these pathways could help develop more effective strategies to treat giardiasis and mitigate the risk of drug resistance.

## Data Availability

The original contributions presented in the study are included in the article/supplementary material, further inquiries can be directed to the corresponding author.

## References

[B1] Adam R. D. (2021). Giardia duodenalis: biology and pathogenesis. *Clin. Microbiol. Rev.* 34 e24–e19. 10.1128/CMR.00024-19 34378955 PMC8404698

[B2] Argüello-García R. Calzada F. García-Hernández N. Chávez-Munguía B. Velázquez-Domínguez J. A. (2020). Ultrastructural and proapoptotic-like effects of kaempferol in Giardia duodenalis trophozoites and bioinformatics prediction of its potential protein target. *Mem. Inst. Oswaldo Cruz* 115 e200127. 10.1590/0074-02760200127 33111756 PMC7577037

[B3] Bagchi S. Oniku A. E. Topping K. Mamhoud Z. N. Paget T. A. (2012). Programmed cell death in Giardia. *Parasitology* 139 894–903. 10.1017/S003118201200011X 22405231

[B4] Benchimol M. Gadelha A. P. de Souza W. (2023). Ultrastructural Alterations of the Human Pathogen Giardia intestinalis after Drug Treatment. *Pathogens* 12 810. 10.3390/pathogens12060810 37375500 PMC10302959

[B5] Caly L. Druce J. D. Catton M. G. Jans D. A. Wagstaff K. M. (2020). The FDA-approved drug ivermectin inhibits the replication of SARS-CoV-2 in vitro. *Antiv. Res.* 178 104787. 10.1016/j.antiviral.2020.104787 32251768 PMC7129059

[B6] Campbell W. C. (2012). History of avermectin and ivermectin, with notes on the history of other macrocyclic lactone antiparasitic agents. *Curr. Pharm. Biotechnol.* 13 853–865. 10.2174/138920112800399095 22039784

[B7] Cheung C. Tu S. Feng Y. Wan C. Ai H. Chen Z. (2024). Mitochondrial quality control dysfunction in osteoarthritis: Mechanisms, therapeutic strategies & future prospects. *Arch. Gerontol. Geriatr.* 125 105522. 10.1016/j.archger.2024.105522 38861889

[B8] Chiumiento L. Bruschi F. (2009). Enzymatic antioxidant systems in helminth parasites. *Parasitol. Res.* 105 593–603. 10.1007/s00436-009-1483-0 19462181

[B9] Crossley R. Marshall J. Clark J. T. Holberton D. V. (1986). Immunocytochemical differentiation of microtubules in the cytoskeleton of Giardia lamblia using monoclonal antibodies to alpha-tubulin and polyclonal antibodies to associated low molecular weight proteins. *J. Cell Sci.* 80 233–252. 10.1242/jcs.80.1.233 3522613

[B10] Dawson S. C. Sagolla M. S. Cande W. Z. (2007). The cenH3 histone variant defines centromeres in Giardia intestinalis. *Chromosoma* 116 175–184. 10.1007/s00412-006-0091-3 17180675

[B11] Elmehy D. A. Hasby Saad M. A. El Maghraby G. M. Arafa M. F. Soliman N. A. Elkaliny H. H. (2021). Niosomal versus nano-crystalline ivermectin against different stages of Trichinella spiralis infection in mice. *Parasitol. Res.* 120 2641–2658. 10.1007/s00436-021-07172-1 33945012

[B12] Fan N. Zhang L. Wang Z. Ding H. Yue Z. (2024). Ivermectin Inhibits Bladder Cancer Cell Growth and Induces Oxidative Stress and DNA Damage. *Anticancer Agents Med. Chem.* 24 348–357. 10.2174/0118715206274095231106042833 38375808

[B13] Farthing M. J. (1997). The molecular pathogenesis of giardiasis. *J. Pediatr. Gastroenterol. Nutr.* 24 79–88. 10.1002/j.1536-4801.1997.tb01456.x 9093992

[B14] Foletto V. R. Vanz F. Gazarini L. Stern C. A. Tonussi C. R. (2015). Efficacy and security of ivermectin given orally to rats naturally infected with *Syphacia* spp. *Giardia* spp. and Hymenolepis nana. *Lab. Anim.* 49 196–200. 10.1177/0023677214562850 25480657

[B15] Foley D. H. Bryan J. H. Lawrence G. W. (2000). The potential of ivermectin to control the malaria vector Anopheles farauti. *Trans. R. Soc. Trop. Med. Hyg.* 94 625–628. 10.1016/S0035-9203(00)90211-6 11198644

[B16] Fraccaroli L. Ruiz M. D. Perdomo V. G. Clausi A. N. Balcazar D. E. Larocca L. (2022). Broadening the spectrum of ivermectin: Its effect on Trypanosoma cruzi and related trypanosomatids. *Front. Cell. Infect. Microbiol.* 12 885268. 10.3389/fcimb.2022.885268 35967842 PMC9366347

[B17] Freitas C. S. Lage D. P. Machado A. S. Vale D. L. Martins V. T. Cardoso J. M. O. (2023). Exploring drug repositioning for leishmaniasis treatment: Ivermectin plus polymeric micelles induce immunological response and protection against tegumentary leishmaniasis. *Cytokine* 164 156143. 10.1016/j.cyto.2023.156143 36774730

[B18] Gadelha A. P. R. Bravim B. Vidal J. Reignault L. C. Cosme B. Huber K. (2019). Alterations on growth and cell organization of Giardia intestinalis trophozoites after treatment with KH-TFMDI, a novel class III histone deacetylase inhibitor. *IJMM* 309 130–142. 10.1016/j.ijmm.2019.01.002 30665874

[B19] Ghosh E. Ghosh A. Ghosh A. N. Nozaki T. Ganguly S. (2009). Oxidative stress-induced cell cycle blockage and a protease-independent programmed cell death in microaerophilic Giardia lamblia. *Drug Design Dev. Therapy* 3 103–110. 10.2147/DDDT.S5270 19920926 PMC2769235

[B20] Gillin F. D. Diamond L. S. (1981). Entamoeba histolytica and Giardia lamblia: growth responses to reducing agents. *Exp. Parasitol.* 51 382–391. 10.1016/0014-4894(81)90125-9 6262103

[B21] Gillin F. D. Hagblom P. Harwood J. Aley S. B. Reiner D. S. McCaffery M. (1990). Isolation and expression of the gene for a major surface protein of Giardia lamblia. *Proc. Natl. Acad. Sci. U. S. A.* 87 4463–4467. 10.1073/pnas.87.12.4463 2352929 PMC54135

[B22] Gillin F. D. Reiner D. S. McCaffery J. M. (1996). Cell biology of the primitive eukaryote Giardia lamblia. *Annu. Rev. Microbiol.* 50 679–705. 10.1146/annurev.micro.50.1.679 8905095

[B23] Gupta S. Vohra S. Sethi K. Gupta S. Bera B. C. Kumar S. (2022). In vitro anti-trypanosomal effect of ivermectin on Trypanosoma evansi by targeting multiple metabolic pathways. *Trop. Anim. Health Prod.* 54 240. 10.1007/s11250-022-03228-1 35869164 PMC9307293

[B24] Hagen K. D. McInally S. G. Hilton N. D. Dawson S. C. (2020). Microtubule organelles in Giardia. *Adv. Parasitol.* 107 25–96. 10.1016/bs.apar.2019.11.001 32122531 PMC8086724

[B25] Hanafi H. A. Szumlas D. E. Fryauff D. J. El-Hossary S. S. Singer G. A. Osman S. G. (2011). Effects of ivermectin on blood-feeding Phlebotomus papatasi, and the promastigote stage of Leishmania major. *Vector Borne Zoonotic Dis.* 11 43–52. 10.1089/vbz.2009.0030 20518644

[B26] Henry C. M. Hollville E. Martin S. J. (2013). Measuring apoptosis by microscopy and flow cytometry. *Methods* 61 90–97. 10.1016/j.ymeth.2013.01.008 23403105

[B27] Houshaymi B. Nasreddine N. Kedees M. Soayfane Z. (2019). Oleic acid increases uptake and decreases the P-gp-mediated efflux of the veterinary anthelmintic Ivermectin. *Drug Res.* 69 173–180. 10.1055/a-0662-5741 30103215

[B28] Joray M. B. Trucco L. D. Gonzalez M. L. Napal G. N. Palacios S. M. Bocco J. L. (2015). Antibacterial and Cytotoxic Activity of Compounds Isolated from Flourensia oolepis. *eCAM* 2015 912484. 10.1155/2015/912484 26819623 PMC4706877

[B29] Katsuno K. Burrows J. N. Duncan K. Hooft, van Huijsduijnen R. Kaneko T. (2015). Hit and lead criteria in drug discovery for infectious diseases of the developing world. *Nat. Rev. Drug Discov.* 14 751–758. 10.1038/nrd4683 26435527

[B30] Katz N. Araujo N. Coelho P. M. Z. Morel C. M. Linde-Arias A. R. Yamada T. (2017). Ivermectin efficacy against Biomphalaria, intermediate host snail vectors of Schistosomiasis. *J. Antibiot.* 70 680–684. 10.1038/ja.2017.31 28293033

[B31] Kho H. P. Leow C. Y. Shueb R. H. Leow C. H. Lim B. H. Chua C. (2018). A hypothetical oxidative stress regulatory role of alpha giardins in the protozoan parasite Giardia intestinalis. *Trop. Biomed.* 35 41–49. 33601775

[B32] Kobylinski K. C. Foy B. D. Richardson J. H. (2012). Ivermectin inhibits the sporogony of Plasmodium falciparum in Anopheles gambiae. *Malaria J.* 11 381. 10.1186/1475-2875-11-381 23171202 PMC3519548

[B33] Kobylinski K. C. Ubalee R. Ponlawat A. Nitatsukprasert C. Phasomkulsolsil S. Wattanakul T. (2017). Ivermectin susceptibility and sporontocidal effect in Greater Mekong Subregion Anopheles. *Malaria J.* 16 280. 10.1186/s12936-017-1923-8 28687086 PMC5501099

[B34] Ledda-Columbano G. M. Coni P. Curto M. Giacomini L. Faa G. Oliverio S. (1991). Induction of two different modes of cell death, apoptosis and necrosis, in rat liver after a single dose of thioacetamide. *Am. J. Pathol.* 139 1099–1109. 1683163 PMC1886348

[B35] Li L. Wang C. C. (2006). A likely molecular basis of the susceptibility of Giardia lamblia towards oxygen. *Mol. Microbiol.* 59 202–211. 10.1111/j.1365-2958.2005.04896.x 16359329

[B36] Liu J. Zhang K. Cheng L. Zhu H. Xu T. (2020). Progress in Understanding the Molecular Mechanisms Underlying the Antitumour Effects of Ivermectin. *Drug Design Dev. Therapy* 14 285–296. 10.2147/DDDT.S237393 32021111 PMC6982461

[B37] Liu Y. Fang S. Sun Q. Liu B. (2016). Anthelmintic drug ivermectin inhibits angiogenesis, growth and survival of glioblastoma through inducing mitochondrial dysfunction and oxidative stress. *Biochem. Biophys. Res. Commun.* 480 415–421. 10.1016/j.bbrc.2016.10.064 27771251

[B38] Lloyd D. Harris J. C. Maroulis S. Biagini G. A. Wadley R. B. Turner M. P. (2000). The microaerophilic flagellate Giardia intestinalis: oxygen and its reaction products collapse membrane potential and cause cytotoxicity. *Microbiology* 146(Pt 12), 3109–3118. 10.1099/00221287-146-12-3109 11101669

[B39] Martinez-Espinosa R. Arguello-Garcia R. Saavedra E. Ortega-Pierres G. (2015). Albendazole induces oxidative stress and DNA damage in the parasitic protozoan Giardia duodenalis. *Front. Microbiol.* 6 800. 10.3389/fmicb.2015.00800 26300866 PMC4526806

[B40] Mastronicola D. Falabella M. Testa F. Pucillo L. P. Teixeira M. Sarti P. (2014). Functional characterization of peroxiredoxins from the human protozoan parasite Giardia intestinalis. *PLoS Negl. Trop. Dis.* 8 e2631. 10.1371/journal.pntd.0002631 24416465 PMC3886907

[B41] Mayol G. F. Revuelta M. V. Salusso A. Touz M. C. Ropolo A. S. (2020). Evidence of nuclear transport mechanisms in the protozoan parasite Giardia lamblia. *Biochim. Biophys. Acta Mol. Cell Res.* 1867 118566. 10.1016/j.bbamcr.2019.118566 31672613

[B42] McKerrow J. H. (2015). Recognition of the role of Natural Products as drugs to treat neglected tropical diseases by the 2015 Nobel prize in physiology or medicine. *Nat Prod. Rep.* 32 1610–1611. 10.1039/C5NP90043C 26510605

[B43] Mosmann T. (1983). Rapid colorimetric assay for cellular growth and survival: application to proliferation and cytotoxicity assays. *J. Immunol. Methods* 65 55–63. 10.1016/0022-1759(83)90303-4 6606682

[B44] Mosquillo M. F. Bilbao L. Hernandez F. Tissot F. Gambino D. Garat B. (2018). Trypanosoma cruzi biochemical changes and cell death induced by an organometallic platinum-based compound. *Chem. Biol. Drug Design* 92 1657–1669. 10.1111/cbdd.13332 29745031

[B45] Mukaratirwa S. Dzoma B. M. Matenga E. Ruziwa S. D. Sacchi L. Pozio E. (2008). Experimental infections of baboons (Papio spp.) and vervet monkeys (*Cercopithecus aethiops*) with Trichinella zimbabwensis and successful treatment with ivermectin. *Onderstepoort J. Vet. Res.* 75 173–180. 10.4102/ojvr.v75i2.16 18788211

[B46] Ordonez-Quiroz A. Ortega-Pierres M. G. Bazan-Tejeda M. L. Bermudez-Cruz R. M. (2018). DNA damage induced by metronidazole in Giardia duodenalis triggers a DNA homologous recombination response. *Exp. Parasitol.* 194 24–31. 10.1016/j.exppara.2018.09.004 30237050

[B47] Park H. Song G. Lim W. (2020). Ivermectin-induced programmed cell death and disruption of mitochondrial membrane potential in bovine mammary gland epithelial cells. *Pestic Biochem. Physiol.* 163 84–93. 10.1016/j.pestbp.2019.10.011 31973874

[B48] Pooda S. H. Mouline K. De Meeus T. Bengaly Z. Solano P. (2013). Decrease in survival and fecundity of Glossina palpalis gambiensis vanderplank 1949 (Diptera: Glossinidae) fed on cattle treated with single doses of ivermectin. *Paras. Vectors* 6 165. 10.1186/1756-3305-6-165 23741989 PMC3679994

[B49] Reis T. A. R. Oliveira-da-Silva J. A. Tavares G. S. V. Mendonca D. V. C. Freitas C. S. Costa R. R. (2021). Ivermectin presents effective and selective antileishmanial activity in vitro and in vivo against Leishmania infantum and is therapeutic against visceral leishmaniasis. *Exp. Parasitol.* 221 108059. 10.1016/j.exppara.2020.108059 33338468

[B50] Rivero M. R. Vranych C. V. Bisbal M. Maletto B. A. Ropolo A. S. Touz M. C. (2010). Adaptor protein 2 regulates receptor-mediated endocytosis and cyst formation in Giardia lamblia. *Biochem. J.* 428 33–45. 10.1042/BJ20100096 20199400 PMC2861151

[B51] Sagolla M. S. Dawson S. C. Mancuso J. J. Cande W. Z. (2006). Three-dimensional analysis of mitosis and cytokinesis in the binucleate parasite Giardia intestinalis. *J. cell Sci.* 119 4889–4900. 10.1242/jcs.03276 17105767

[B52] Schindelin J. Arganda-Carreras I. Frise E. Kaynig V. Longair M. Pietzsch T. (2012). Fiji: an open-source platform for biological-image analysis. *Nat. Methods* 9 676–682. 10.1038/nmeth.2019 22743772 PMC3855844

[B53] Soliman G. A. Taher E. S. Mahmoud M. A. (2011). Therapeutic efficacy of Dormectin, Ivermectin and Levamisole against different stages of Trichinella spiralis in rats. *Turk. Parazitol. Derg.* 35 86–91. 10.5152/tpd.2011.22 21776593

[B54] Sulik M. Antoszczak M. Huczynski A. Steverding D. (2023). Antiparasitic activity of ivermectin: Four decades of research into a “wonder drug”. *Eur. J. Med. Chem.* 261 115838. 10.1016/j.ejmech.2023.115838 37793327

[B55] Taylor R. C. Cullen S. P. Martin S. J. (2008). Apoptosis: controlled demolition at the cellular level. *Nat. Rev. Mol. Cell Biol.* 9 231–241. 10.1038/nrm2312 18073771

[B56] Udensi U. K. Fagbenro-Beyioku A. F. (2012). Effect of ivermectin on Trypanosoma brucei brucei in experimentally infected mice. *J. Vector Borne Dis.* 49 143–150. 10.4103/0972-9062.213454 23135008

[B57] Upcroft P. Upcroft J. A. (2001). Drug targets and mechanisms of resistance in the anaerobic protozoa. *Clin. Microbiol. Rev.* 14 150–164. 10.1128/CMR.14.1.150-164.2001 11148007 PMC88967

[B58] Vicente B. Lopez-Aban J. Chaccour J. Hernandez-Goenaga J. Nicolas P. Fernandez-Soto P. (2021). The effect of ivermectin alone and in combination with cobicistat or elacridar in experimental Schistosoma mansoni infection in mice. *Sci. Rep.* 11 4476. 10.1038/s41598-021-84009-y 33627744 PMC7904857

